# Courage, hope, and transformation: Patient experiences of web-based, therapist delivered eye movement desensitization and reprocessing for suicidal ideation

**DOI:** 10.1371/journal.pmen.0000373

**Published:** 2025-08-07

**Authors:** Sidney Yap, Manroop Bal, Mina Youakim, Ashraf Aborawi, Genna Di Pinto, Krystle Samways, Amy Beck, Katherine S. Bright, Suzette Brémault-Phillips, Andrew J. Greenshaw, Olga Winkler, Lisa Burback

**Affiliations:** 1 Department of Psychiatry, University of Alberta, Edmonton, Alberta, Canada; 2 Heroes in Mind, Advocacy and Research Consortium, University of Alberta, Edmonton, Alberta, Canada; 3 Department of Occupational Therapy, University of Alberta, Edmonton, Alberta, Canada; 4 School of Nursing, Thompson Rivers University, Kamloops, British Columbia, Canada; 5 School of Nursing and Midwifery, Mount Royal University, Calgary, Alberta, Canada; 6 Alberta Hospital Edmonton, Edmonton, Alberta, Canada; 7 Neuroscience and Mental Health Institute, University of Alberta, Edmonton, Alberta, Canada; University of Luxembourg: Universite du Luxembourg, LUXEMBOURG

## Abstract

Suicide is a significant global public health issue. Existing evidence-based interventions for suicidal ideation (SI), while helpful, have limitations including suboptimal efficacy, and high resource intensiveness and attrition. As SI is often related to stressful events, Eye Movement Desensitization and Reprocessing (EMDR) psychotherapy, an evidence-based treatment for posttraumatic stress disorder (PTSD), has been utilized as a novel approach for addressing SI. This study explored the lived experiences of adult mental health outpatients who received web-based, therapist-delivered EMDR targeting experiences related to their SI. This qualitative study, which is part of a larger randomized controlled trial (RCT) investigating web-based EMDR for adults with SI, recruited participants from among those who received EMDR treatment. Researchers collected data using semi-structured interviews over Zoom and conducted initial thematic descriptive analysis and coding guided by Braun and Clarke’s thematic analysis framework. Fourteen EMDR treatment group participants agreed to be interviewed. Five main themes were identified: (1) systemic obstacles to recovery, (2) relational attunement creating safety, (3) moving past the stuckness of suicidal states, (4) posttraumatic growth and resilience, and (5) unique needs and preferences. Most study participants reported that web-based EMDR therapy for SI was an overall beneficial experience, noting that a strong therapeutic relationship and ability to personalize treatment were key for treatment success. The encouraging and predominantly positive insights from participants in this pioneering work established a foundation for future research and clinical practice regarding the application of EMDR for SI.

## Introduction

Suicide is a well-recognized global public health problem, with the World Health Organization estimating that more than 700,000 people die by suicide every year [1]. While certain medications have modest anti-suicidal properties [2], the mainstay of treatment involves psychotherapeutic approaches [3]. Current evidence-based, gold-standard psychotherapeutic interventions for suicidal ideation (SI) include Cognitive Behavior Therapy (CBT) based interventions, Dialectical Behavior Therapy (DBT), and Crisis Response Planning (CRP). These interventions generally focus on teaching coping and emotional regulation skills. While helpful for many, such interventions have significant limitations, including modest effectiveness for reducing SI and suicidal behaviors, high resource-intensivity, and high attrition [4].

Trauma-focused psychotherapy (TFP) may be a promising alternative approach for addressing SI. First, exposure to psychological trauma (e.g., maltreatment, family violence) is highly associated with development of SI [5], and TFP treatment of posttraumatic stress disorder (PTSD) is generally associated with reductions in SI [6,7]. In addition, childhood or recurrent trauma may impact social, personality, and brain development, increasing the risk of impulsivity, maladaptive coping, and emotional dysregulation, which are associated with suicidality [8]. Second, according to current suicide theories, SI is viewed as a perceived solution for inescapable pain, entrapment, perceived burdensomeness, thwarted belongingness, or hopelessness originating in overwhelming experiences such as trauma, loss, humiliation, or rejection [9–11]. Rudd proposed that such experiences can contribute to the development of patterned suicidal states with emotional, cognitive, sensorimotor, and physiological components (e.g., urges, sensations, arousal) that can be recurrently activated by future stressors [12]. Suicidal states can thus act as latent, enduring, and evolving chronic risk factors for future suicidality after the original precipitating events have passed [12]. From a trauma-informed perspective, these suicidal states may be conceptualized as arising from implicit and explicit memories amenable to TFPs [13]. Addressing suicidal states through TFP treatment may therefore have the potential to decrease SI [14]. Psychotherapeutic treatment may also provide other concomitant benefits to patients, including instilling hope, facilitating emotional processing, and improving insight [15], all of which may aid in the resolution of suicidal states.

Eye Movement Desensitization and Reprocessing (EMDR), an evidence-based TFP for PTSD [16], has recently emerged as a potential treatment option for lowering acute and chronic SI in addition to addressing PTSD [7,17–20]. Recent research has found that EMDR treatment may lead to improvements in SI as an outcome of treating specific psychiatric diagnoses, such as major depressive disorder [18] and PTSD [7] and aid in the processing of traumatic memories or events clearly linked to the development of SI [20]. Researchers have also explored the potential of EMDR for suicidal intrusions [19] and borderline personality disorder (BPD) [17,21].

Recognizing this potential, our research group conducted a randomized controlled trial (RCT) assessing the effects of 12 sessions of web-based, synchronous therapist-delivered EMDR for SI. The treatment focused on memories, beliefs, and other symptoms associated with suicidal states rather than on a specific psychiatric disorder [22]. Despite enrolling a population with high trauma load, longstanding SI, and high levels of psychiatric comorbidity, quantitative results were encouraging [22]. Participants reported significant decreases in SI and symptoms of depression, anxiety, and PTSD. EMDR group participants also had fewer serious adverse events (e.g., emergency room visits, hospitalizations) compared to treatment as usual (TAU) group participants [22].

Given the novel treatment approach, we sought to capture data regarding participant lived experiences and recommendations related to receiving web-based EMDR focused on SI. Exploring patient experiences, alongside quantitative measures of clinical effectiveness and patient safety, has been recognized as an important aspect of healthcare quality [23–25], particularly in the context of patient-centered care. The breadth and nuance of such experiences are typically lost in quantitative studies and require qualitative approaches. There is, however, a general paucity of qualitative studies related to participant experiences of EMDR therapy [26], and, to our knowledge, no such EMDR studies in populations experiencing suicidal states specifically. The provision of TFPs to those with SI is often avoided due to clinician concerns related to patient safety and symptom exacerbation [6,27,28]. In addition, few studies exist related to patient EMDR experience in the context of web-based delivery. Such research is crucial for ensuring interventions are relevant for and accepted by real-world clinical populations and can be adapted to individual needs [29].

The aim of this qualitative study was to explore the experiences of adult psychiatric outpatients who received web-based, therapist-delivered EMDR targeting suicidal state-related content. We anticipated that these findings would deepen our understanding of participant’s priorities when receiving web-based EMDR treatment and capture important patient-driven recommendations for future improvements.

## Methods

This qualitative study employed individual semi-structured interviews to explore the experiences of adult psychiatric outpatients who received web-based, therapist-delivered EMDR. The study was conceptualized following commencement of participant recruitment and data collection in a larger *Randomized Controlled Trial Investigating Virtual Eye Movement Desensitization and Reprocessing for Adults with Suicidal Ideation* (protocol ID number: Pro00090989) registered on ClinicalTrials.gov (ID number: NCT04181047). As such, this qualitative component was added to the RCT through an ethics amendment and approved by the University of Alberta Health Research Ethics Board prior to commencing research activities.

### Ethics statement

This study followed the ethical guidelines outlined in the Declaration of Helsinki. The University of Alberta Research Ethics Board approved this study prior to the commencement of research activities (protocol ID number: Pro00090989). Informed consent was obtained from all participants prior to study participation. This involved providing the participants with an informed consent form that detailed the purpose of the study, potential risks and benefits, confidentiality measures, and their rights as study participants. This form was presented in written format, and participants were encouraged to ask questions before agreeing to participate in the study. A research team member was present to confirm that the participant understood the information within the consent form and voluntarily consented to being a part of the study. This research team member also documented and verified the authenticity of the participants’ consent.

### Participant recruitment and eligibility

Participants were recruited from the EMDR treatment group of the *Randomized Controlled Trial Investigating Virtual Eye Movement Desensitization and Reprocessing for Adults with Suicidal Ideation* [12]. Inclusion criteria for the parent clinical trial included being between 18–65 years of age, reporting SI in the past week, having an established and ongoing relationship with another healthcare provider outside the study, having access to appropriate technology and space for web-based therapy (e.g., a working computer and a private space for sessions), and committing to twice weekly EMDR sessions for the study duration. Those with imminent suicidal plans or severe dissociative symptoms (i.e., Dissociative Experiences Scale II (DES-II) score above 34 or symptoms indicating a dissociative disorder on clinical assessment) were excluded from the study. Examples of severe dissociative symptoms meeting exclusion criteria included dissociative voices, amnestic episodes, dissociative fugue states, passivity experiences or first rank symptoms under stress. Participant recruitment for the parent RCT began on May 1, 2021, and concluded on June 30, 2023.

Participants were contacted by their study therapist at least two weeks after their final EMDR treatment to be invited to participate in this qualitative study. All EMDR treatment group participants, including dropouts, were contacted. Interested participants were contacted by an independent interview team to provide study information and obtain informed consent electronically through Research Electronic Data Capture (REDCap) [30]. An interview session was subsequently scheduled. Participant recruitment for the current study began on January 9, 2023, and concluded on July 31, 2023.

### Semi-structured interviews

Individual 30–60-minute semi-structured interviews were conducted and video-recorded over Zoom [31] between January 27, 2023, and August 8, 2023. Interviews were led by a two-person team consisting of an experienced clinician (a registered psychologist and psychiatric resident) and a research assistant. Individual interviews were chosen over focus groups due to the personal and potentially sensitive nature of the topics discussed [32]. Prior to the start of these qualitative interviews, a safety plan was formulated to manage potential situations wherein a participant might disclose psychological distress requiring crisis intervention or clinical follow up.

Interview guides were developed by the research team in collaboration with the clinicians who delivered EMDR in the parent RCT. Key topics included describing the participants’ experience of receiving web-based EMDR for SI, whether any changes occurred as a result, and any aspects they wished had been different about their participation. See Appendix 1 for a copy of the interview guide (Appendix 1).

### Data analysis

Interviews were transcribed verbatim using Adobe Premiere Pro, with transcription accuracy checked by a research team member (SY, MY, AA, GDP, KS), and analyzed using NVivo 14. Transcripts were subjected to inductive thematic descriptive analysis following an iterative process without imposing a pre-existing coding frame [33]. This process involved identifying emerging themes from the data.

Two of six research team members (SY, MB, MY, AA, GDP, and KS) independently coded each interview. Following initial coding, a group of senior researchers (SBP, AB, and KB) reviewed and refined the codes. Preliminary themes and supporting quotes were then identified and tabulated. The senior research team (SY, SBP, AB, KB, OW, LB) then analyzed and modified the preliminary themes, resolving differences through discussion and isolated key quotes. The final thematic narrative was then prepared. The Standard for Reporting Qualitative Research was used to guide the reporting process [34].

The clinician researchers who administered web-based EMDR treatment in *Randomized controlled trial investigating virtual Eye Movement Desensitization and Reprocessing for adults with suicidal ideation* (Pro00090989) were kept at an arm’s length during interviews and initial analysis. This minimized potential bias from the clinician researchers and helped maintain participant’s anonymity and privacy.

## Results

### Participant demographics

Of the 20 EMDR treatment group participants from the parent clinical trial, 14 agreed to be interviewed. Owing to the highly unique perspectives shared by study participants, 14 interviews were deemed to be sufficient for reaching adequate information power [35]. Twelve of the 14 participants in the current study completed 12-sessions of web-based EMDR, while the remaining two participants completed two and four EMDR sessions, respectively. Interviews were conducted after an average of 41 weeks (range: 3–84 weeks) had elapsed following treatment completion. Participants were an average age of 37 ± 13.6 years (range: 18–60 years old). Nine participants self-identified as women, and five as men, and none identified as transgender or gender diverse.

Eight of the 14 (57%) participants who completed an interview presented with at least three Diagnostic and Statistical Manual of Mental Disorders-5 (DSM-5) diagnoses. The most common diagnoses were mood disorders (n = 12, 86%), anxiety disorders (n = 10, 71%), and trauma-related disorders (n = 7, 50%). In addition, seven participants (50%) had either strong traits or a full diagnosis of BPD (BPD: n = 4 (29%); BPD traits: n = 3 (21%)). The median age of ﬁrst experiencing SI was 13 years (range: 7–50 years old), while median suicide attempts was 1 (range: 0–20). The demographic information for all EMDR treatment group participants in the parent clinical trial can be found in Burback et al., 2024 [22].

### Interview results

Descriptive analysis of interview transcripts isolated five main themes and 17 sub-themes. The five main themes include: (1) systemic obstacles to recovery, (2) relational attunement creating safety, (3) moving past the stuckness of suicidal states, (4) posttraumatic growth and resilience, and (5) unique needs and preferences.

#### *Theme one*: Systemic obstacles to recovery.

Prior to commencing EMDR in this study, many participants expressed encountering significant systemic barriers when trying to access mental healthcare. They underscored the need for improvements in treatment accessibility, responsiveness, and sensitivity to the needs of individuals with SI seeking support. Participants maintained that addressing these barriers would require health system changes that prioritize timely access to care, validate individual experiences, and foster effective collaboration between providers and patients in treatment planning and delivery. See Table 1 for sub-themes and supporting quotes.

**Table 1:** Systemic obstacles to recovery.

Sub-theme	Description and Supportive Quotes
1.1 Difficulty accessing services while in distress	Barriers included limited therapist availability and access to clinics, and poor relationships with therapists. These led to delays in care, exacerbating feelings of hopelessness and discouragement. *“[Waiting] months and months and months[,] I don’t think that would work. I think I would have attempted suicide again. I would have to wait a long, long time[...] And people, like, in my situation, we don’t really have a long time.” - Participant 12* *“If I go back into depression or suicidal thoughts and things like that, I’m a bit worried at how I will deal with that or if I will remember techniques or methods or what to do to recover from that, it’s like, you know, I’m on my own if I can’t access help.” - Participant 14*
1.2 Harm of misattunement and gaslighting by mental health supports	Some highlighted negative prior experiences within the healthcare system, including being dismissed by healthcare professionals and feeling that the root cause of their issues was not sufficiently addressed. A secure, heartfelt connection had been missing for many participants when they had previously received psychotherapy. *“I have [had] a lot of bad therapists in the past that have not been able to help me. So it’s been a little bit of a nightmare.” - Participant 14* *“So it’s funny because they always tell you [to] advocate for yourself and [say how I’m feeling is] not a joke. [You] have to ask over and over and over again, [like] with this specific instance where I was sick, I went to five different E.R. visits because I felt like I couldn’t function at all [...] And I was a mess. And there were times where I was told that I was a burden to the system and it was so discouraging. And it took so long for me to get the help, get into an ER and say, ‘if you send me away, I’m going to die tonight.’” - Participant 2*
1.3 Self-advocacy falling on deaf ears	Some participants felt their self-advocacy for treatment was not aiding in progressing their therapeutic journey, leaving them unsupported and isolated. *“I have complex PTSD (cPTSD) and PTSD [and] I don’t tend to fit into the groups where they would put me with like alcoholics or drug users or anger management because I’m the opposite [..] I grew up as the victim of those people and I’m submissive[...] So to put me in those groups was just adding to the problem [...] But it took a lot of advocating to get to that point [...] I can’t I can’t do this anymore [...]” - Participant 2*

#### *Theme two:* Relational attunement creating safety.

During EMDR treatment, a strong and supportive therapeutic relationship was reported by most participants as foundational for successful EMDR treatment, helping many to open up about their past traumas and connect with their emotions. This emphasizes the essential role of trust, compassion, and collaboration between therapists and patients as important treatment facilitators. See Table 2 for sub-themes and supporting quotes.

**Table 2:** Relational attunement creating safety.

Subtheme	Description and Supportive Quotes
2.1 Accepting and validating therapist	The therapists’ professional, empathetic, and understanding approach fostered trust, creating a safe space for participants to be vulnerable, tolerate distress, and process trauma or distressing past experiences. *“[My clinician] was one of the very first people that I could actually sit with and talk about something that was frightening and unimaginable. And she listened to everything that I said and didn’t tell me I was wrong, or that maybe I should look at it this way or right. (...) [H]er instructions were so clear it was easy to follow and it felt [okay], even though it’s so painful.” - Participant 2* *“I think [my clinician] was more patient and affirming than the first therapist I had.” - Participant 13* *“It was hard at first. Yeah, but I felt like it was, really like it was a kind of progression with [my clinician] (...) because she’s very professional, very empathetic, very understanding and very sensitive and I think that helped a lot.” - Participant 14*
2.2 Feeling safe enough to deepen the therapeutic process	Feeling safe, heard, validated, and well-supported encouraged participants to become vulnerable and engage more deeply in the therapeutic process. *“Like having a back-and-forth conversation. Not just me sitting there listening or them sitting there listening” – Participant 5* *“I kind of couldn’t wait to get back and talk with [my therapist]. It felt like [what was bothering me] was opened, and I just wanted to keep going” - Participant 7*
2.3 Balancing nurturing and nudging	Therapists were tasked with finding the delicate balance between pushing participants’ boundaries while also creating and maintaining a safe and nurturing therapeutic environment. *“And [my clinician was] actually the first person who’s really tried to push [me]. Okay, you need to work through this specifically. Like every other therapist I’ve ever seen has been like, surface level things like, self-esteem. Like, ‘how are you feeling today?’ Not [really getting at] what’s making me feel this.” - Participant 6* *“I seemed to feel better after each session I [had]. I think there were ten or twelve sessions, I can’t remember. But they seemed to bring me to a calmer state. I wanted them to go longer [laughter], when [my clinician] was doing them [...] It’s like having a security blanket, something to look forward to every week that would, ‘oh, I’m going to have another session, maybe I’ll get a little bit better.’” - Participant 8*

#### *Theme three:* Moving past the stuckness of suicidal states.

Participants described the EMDR process as a challenging but rewarding journey, where they confronted distressing memories and recurrent themes, leading to a sense of progress and capacity to cope. Many participants reported an evolution in the quality of their suicidal states and associated ideation and a sense of resolving “root issues”. See Table 3 for sub-themes and supporting quotes.

**Table 3:** Moving past the stuckness of suicidal states.

Sub-theme	Description and Supportive Quotes
3.1 Evolution of suicidal states	Suicidal thoughts moved from active to passive SI, with greater ability to feel and release emotions. *“(My suicidal state) now, it’s more like, okay, thinking about it, what’s going to happen? You know, how is it going to impact everybody? And then I could start to release those emotions, like, you know, the sadness, the anger, the just all the different emotions that come with it.” - Participant 5* *“Despite still struggling with like emotional regulation a bit and a bit of anxiety and depression, but like the suicidal thoughts have like completely disappeared. And I just find that like, so crazy, which I mean, obviously it’s awesome, but yeah, and then in terms of the self-harm as well, like I’ve maybe only thought about it once within the last year, so that’s a huge improvement. And it’s now, it’s like a thought, but there’s no intent behind it.” - Participant 10* *“They (suicidal thoughts) [are] more passive. I do, I do think, I do have thoughts that, you know, sometimes my life is not worth living. And so I wish that I would, you know, go to sleep and not wake up. But, but I’m not actively like planning a suicide” - Participant 14*
3.2 Benefits of addressing root causes of suicidal states	EMDR facilitated exploration of underlying factors contributing to SI, such as unresolved trauma or feelings of disconnection, leading to a sense of progress. *“EMDR was the first time where I actually gained some ground because it was working on the root issues that had caused me to be [this] way [...] I think that it was fantastic and it’s a good fit for somebody who has had enduring child abuse going into adulthood.” - Participant 2* *“Of all the things that I’ve done mental health wise, it was one of the things that helped me the most. And the reason I say that is it helped kind of put stuff to rest that would always brew back up and, you know, affect my mood, affect my suicidal thoughts.” - Participant 12* *“[It] was the first time I’ve ever talked to anybody about that. And I think that it helped me desensitize to it because before EMDR, like, I couldn’t even, like even the thought of it or if somebody like, whenever I started talking about it, I would just freeze up. With this EMDR and particularly [my clinician], I was able to take a deep dive into things that I’ve never talked to anybody else about, I’ve never shared with anybody else before. So [I was able to get] into the real root of things.” - Participant 14*
3.3 Acquisition of coping strategies	EMDR enabled participants to better cope with and navigate through waves emotional turmoil and suicidal states. *“[My clinician] taught me, like, tapping my shoulder or tapping my knees. Um, and it’s just helpful doing something physical that isn’t -- that isn’t hurting myself [laughter] to kind of take me out of an anxiety attack, or just a big emotion to, yeah kind of ride the wave [...] And then she would say, you know, like, oh, today because we did this, maybe go for a walk, kind of get moving or, or just relax for a while. Like, she would always give me recommendations on how to recover after.” - Participant 4* *“I’ve taken my safe place from the EMDR that I did with [my clinician] to the EMDR that I’m doing now. So I have my safe place [...] I’ve got some ways of practicing meditation, those kinds of things, just to make breathing techniques [...] instead of falling apart, now I have a little bit of tools to use.” - Participant 7* *“[My clinician would remind me] to do the container and to put things away at bedtime. There’s a huge difference between whether I do that or if I [forgot]. So at bedtime, I need to make sure that everything is put away in the box. The parts are all in their safe places.” - Participant 2*

#### *Theme four:* Posttraumatic growth and resilience.

Throughout the EMDR process, participants found hope for future recovery as they began to navigate daily challenges with deepened self-awareness, compassion, and acceptance, likely aiding in reducing SI. Resulting changes included increased engagement in social activities, securing and maintaining new employment, improving tenuous relationships with family members, and increased self-compassion. See Table 4 for sub-themes and supporting quotes.

**Table 4:** Posttraumatic growth and resilience.

Sub-theme	Description and Supportive Quotes
4.1 Self-compassion	EMDR therapy facilitated a deeper knowing of oneself leading to increased self-compassion. *“It did help me gain a bit more self-love, I would say, even just like -- because we did have, you know, like thinking of all the different versions of myself, I can think of, you know, an eight-year-old version of me that I can have a lot of love for.” - Participant 4* *“I feel like I’m happier. Um, no real reason. Just feeling a little bit more free, and, um, that there was closure in a lot of areas that we worked on. I know that I blame myself for a lot of the things that happened, and with EMDR, it has allowed me to get past that. That I’m not at fault for anything.” - Participant 7*
4.2 Hope for the future	Despite difficulties, participants reflected an increase in hope for recovery and their future, contributing to a reduction in suicidal intent. *“So I guess, who knows, maybe EMDR did contribute to me finally being like, I don’t want to die. I want to live a long, healthy life and figure this out.” - Participant 1* *“But the EMDR definitely brought a little more new light to me there, which showed me there are new treatments coming out. You know, it’s okay to have that hope and to just keep pushing forward until I find out what works for me [...] I’m not the only one who feels this way [, I just] need to keep pushing and just keep going, but don’t quit.” - Participant 5* *“I’ve kind of muted the thoughts almost, because I realized like -- I kind of have no choice but to just continue navigating life because I’ve overcome everything that has come my way so far.” - Participant 10*
4.3 Enhanced interpersonal relationships and functioning	Some participants reported improved communication with their partners and children, enhancing their interpersonal relationships. They also experienced better reintegration into life, including improved social functioning and/or employment. *“I noticed it was a lot easier to communicate with my husband -- just to be a more effective communicator, more open and honest, [as] anxiety will get the better of me sometimes. And I’ll just say, or think, hold things back to not sound like an idiot [...] so taking the anxiety out of it has made communicating a lot better and, and with the kids, just, a lot easier to -- I guess like relax into spending time with them, instead of being distracted [or] worrying instead of what I should be doing, which is connecting with my kids, I would -- I think I was pretty disconnected beforehand. And now it’s -- I’m noticing little improvements all the time with our relationships.” - Participant 3* *“Yes, everything’s gotten better [since receiving EMDR treatment]. I’ve gone back to work and social wise, I’m still -- like I can do it. We went to a wedding this Saturday. And I’m not a hermit, but I pick and choose what kind of social setting I’ll put myself in.” - Participant 12* *“There’s one time when I was still pretty sick, my little girl says, we had a death in the family and she just said to my husband very nonchalantly “I think mommy is going to die first because she’s always sick”[...] She recognizes that her mom is always sick and that’s, that’s crappy. But now [after EMDR I noticed that] she doesn’t say things like that, that stuff doesn’t come up. So I think [...] there is a positive change because those conversations or those comments aren’t happening.” - Participant 2*

#### *Theme five:* Unique needs and preferences.

This theme highlights the nuanced nature of participants’ personal preferences regarding EMDR therapy. Participants had varying suggestions for improving future EMDR delivery, likely reflecting their diverse underlying issues and variable readiness to engage in therapy. Whereas some expressed biweekly sessions led to emotional exhaustion, others desired even more frequent EMDR sessions. Additionally, the overall duration of treatment was either not commented on or believed to be too short. Finally, some participants appreciated the online delivery format while others found it too impersonal. See Table 5 for sub-themes and supporting quotes.

**Table 5:** Unique needs and preferences.

Sub-theme	Description and Supportive Quotes
5.1 Pacing, frequency, and intensity of sessions	While some participants found the pacing of sessions appropriate, others desired a different session schedule. For example, some participants experienced strong mental and emotional fatigue after EMDR sessions and wanted more time between sessions. Other participants, in contrast, expressed an interest in having more frequent sessions. *“I would almost rather do seven days of intense EMDR therapy all together so that I don’t have to do the constant up and down and up and down [...] I almost feel like that would be more effective because it is so exhausting that I would almost rather have 3 to 6 weeks of crazy exhaustion and over-stimulation than months and months and months of drawing out the pain in little pieces.” - Participant 2* *“The frequency felt good. We did it twice a week, back-to-back. And the second one was usually a lot more tiring, having been the second one in a row. But the frequency was good, I thought. Yeah, the length of the study was good. I felt like I got lots of positive changes out of the amount of EMDR that I had.” - Participant 3* *“And it’s like, 2 hours a session, which [is] like 2 hours, or I guess 4 hours a week, is a lot for a working adult.” - Participant 4* *“I found it kind of stressful or emotionally draining each time I had a session. [We] started out doing twice a week and I found that was too much for me. Like there was one or two days where I ended up just not going to school afterwards. I just felt really drained. So we ended up switching it to do once a week. And that was all right for me.” - Participant 13*
5.2 Treatment duration	Some participants felt that the treatment course overall was too short, hoping for more EMDR sessions after study completion to continue to work on their suicidal states. Conversely, no participants felt that the treatment duration was too long. *“I was hoping for -- well, at that time, I was hoping for more sessions. Like, the one-hour sessions were good, but at the time, I wanted them to continue because they were a security thing.” - Participant 11* *“I wish I could have gone on a little bit longer, but I don’t know if that’s feasible in a study. Like the real sessions themselves, not the preparation work that you have to do.” - Participant 12* *“It’s kind of like, from your point of view, there are still some things that you could have explored if you had more sessions.” - Participant 14*
5.3 Readiness for EMDR	Some participants recognized missed opportunities for deeper engagement with EMDR therapy due to personal circumstances and readiness. These participants acknowledged that a different mindset or approach might have yielded more significant therapeutic improvements, highlighting the importance of timing and readiness in therapy outcomes. *“Overall, I think EMDR could have been really helpful for me, but I was not in a position to fully accept that help. So if I were to do it over again, I think I would see a lot more results. But unfortunately, where I was at -- at that time in my life, I just -- I didn’t take advantage of it. I didn’t use it to the full extent that it could have been. And so, unfortunately, it didn’t it didn’t benefit me at the time.” - Participant 6* *“When I was going through the process, I don’t know if I was, I just felt I wasn’t seeing, or it wasn’t, I don’t know how to explain, but when we would do [EMDR], I just wouldn’t see anything. So it was very hard to get anywhere or to move forward in the process.” - Participant 8*
5.4 Differing opinions on web-based delivery	Preferences for web-based versus in-person therapy varied, and while online therapy increased accessibility, it also presented challenges related to technology access and knowledge, as well as the therapeutic relationship. *“I was saying to [my clinician] that doing the online part for me was one of the reasons I could even do it. Having the physical distance, it made me a lot more comfortable to really let go and get into it. I also had mentioned to her that because of the way the cameras lined up, I knew she was looking at me in the camera, but it didn’t feel like her eyes were looking into my eyes, which also made me a lot more comfortable. I struggle with that sometimes.” - Participant 3* *“The only other apprehension I have of online stuff -- like even this morning on the laptop that I was using when I was doing my work with [my clinician], I was looking for Zoom and like I hadn’t done a Zoom session on that computer since [my clinician] and I worked together [...] So that part causes me anxiety, the computer part.” - Participant 12* *“[Being online] didn’t feel personal enough. [It] felt more like just watching a video than actually doing therapy with somebody.” - Participant 5* *“But at the time I just wanted human connection, I guess, because we had been in the pandemic for so long and it was still going on and stuff like that.” - Participant 6*
5.5 The complex and bumpy road to recovery	While EMDR yielded positive end outcomes for many, some experienced difficulties during treatment. For these individuals, the complexity of the therapeutic content made it difficult for them to quantify their week-to-week progress. *“There were times after sessions where I ended up feeling worse. It wasn’t every time, but I definitely felt it after at least a couple of sessions[...] And the suicidal ideation -- it wasn’t as prominent, but I’m not sure if that was because of the EMDR or just because my depression went kind of into remission or whatever.” - Participant 6* *“Well, if you want to talk about the therapy itself, if the goal of the [EMDR] was to reduce suicidal thoughts, well, it was a big failure in that way.” - Participant 9*

## Discussion

This study aimed to explore the experiences of adult psychiatric outpatients who received web-based, therapist-delivered EMDR targeting SI. Most participants viewed the intervention positively while also noting it to be intensely emotional. Many emphasized readiness, treatment individualization, and a safe therapeutic relationship as important factors for treatment success. This study contributes to a relatively neglected area of research. Few qualitative studies have explored participant experiences of receiving TFPs generally [36–38], or in-person EMDR [26,39,40] or web-based EMDR specifically [41]. Further, the dearth of qualitative research that includes complex psychiatric populations limits the generalizability of the current literature to those with SI.

To our knowledge, this is the first study to report on (1) the lived experiences of suicidal adult psychiatric outpatients receiving web-based EMDR, and (2) patient experiences of EMDR used to specifically address SI. Given population heterogeneity and the perceived risks of conducting research with such populations, individuals experiencing severe psychiatric illness, comorbidities, and SI have historically been excluded from research [42,43]. The EMDR study protocol employed in this study intentionally sampled a real-world population and allowed for treatment individualization. As a result, study findings may reflect a more realistic representation of how EMDR treatment might be delivered in a clinical setting [22].

While the inclusion of complex psychiatric populations in research has started to become more common [40,44], substantially more research that prioritizes the inclusion of real-world complex clinical populations is needed to sufficiently address the multitude of barriers facing these individuals, such as difficulties accessing timely care and developing strong and supportive therapeutic relationships. For example, finding appropriate mental health services was a commonly described challenge for many participants. Other notable challenges included a lack of accessible and effective treatment options and a lack of validation and support from healthcare professionals with whom they had previously worked with. These and other systemic factors have also been cited in previous research, including limited financial resources, a lack of available treatments, problems securing transportation, and the inconvenience of attending treatment, as barriers to suicidal individuals accessing mental health care [44–46]. Such challenges regrettably contribute to reduced help seeking by many individuals with SI [47]. Online interventions, such as the web-based EMDR offered in this study, provide a promising solution to addressing some concerns regarding the accessibility of current SI treatments [48].

Uniquely, preliminary findings from this qualitative investigation supports the feasibility and safety of web-based EMDR as a transdiagnostic treatment in individuals experiencing SI and a range of concomitant symptoms. While yet to be rigorously tested, reported changes in suicidal states following EMDR align with the Adaptive Information Processing (AIP) model [49,50] and our hypothesis that EMDR can address the unique memory-based constituents of individuals’ suicidal states [12] and a range of psychiatric symptoms [22]. The AIP model suggests that the processing of maladaptively stored memories leads to both symptom reduction and the resolution of barriers to subsequent healing and growth [49,50]. Since posttraumatic presentations may depend on trauma type and burden, the context of the trauma, and attachment experiences [51–54], EMDR may offer the capacity to address individual challenges. Given evidence of PTSD subtypes associated with specific trauma types, future research may explore if there are subtypes of SI associated with specific experiences and circumstances (e.g., life threat, loss, abandonment/rejection, moral injury, humiliation) that create unique suicidal states [55,56].While further research is needed to confirm our results, the findings of this study provide exciting evidence of the multifaceted potential of web-based EMDR.

The perceived effectiveness of the short web-based EMDR treatment by study participants was the most promising of study findings. Participants shared that EMDR was transformative and resulted in changes in their SI (e.g., active to passive SI), citing a sense of notable progress over the 12-session EMDR treatment course. Attributing this progress to addressing the “root” causes of their SI and their sense of “stuckness”, participants were able to more freely speak about their struggles and previous traumas without this inciting a sense of helplessness, loss of control, or dissociative or freeze trauma responses [57]. This may have allowed them to leverage the adaptive coping strategies they had developed throughout their therapeutic journey. Similar findings of becoming “unstuck” have been described by those receiving other TFPs, including cognitive processing therapy (CPT) [58] and Multi-modal Motion-assisted Memory Desensitization and Reconsolidation therapy (3MDR) [59].

Posttraumatic growth (PTG) was commonly experienced by study participants. In alignment with other studies, many study participants showed signs of positive psychological changes following their struggle with trauma or highly challenging situations [60]. While 12 EMDR sessions was likely not enough to fully address participants’ SI-associated memories and suicidal states, reported changes were nonetheless consistent with PTG. These included improvements in self-perception, interpersonal relationships, and philosophy of life, which led to greater self-awareness and self-confidence, a more open attitude towards others, a greater appreciation for life, and the discovery of new possibilities [61,62]. Posttraumatic growth also manifested itself in positive social (e.g., improved relationships with family members), occupational (e.g., finding new employment), and personal (e.g., enhanced emotion regulation) changes following EMDR. Similar increases in hope, future orientation, and functioning have been described in both EMDR [26,40] and TFP [44,59] literature, speaking to the potential of such psychotherapies.

The path to recovery was neither simple, straightforward, nor uniform. While some participants experienced treatment failure or major difficulties during study participation, others described recovery and hope for the future. This variability in treatment outcome is reflected in the TFP literature, with research both supporting [21,63,64] and cautioning against [65] the use of intensive TFP to treat complex psychiatric populations. Accordingly, fears of symptom exacerbation, poor treatment response, and increased treatment drop-out has likely played a role in driving clinician hesitancy to provide intensive TFP, such as EMDR, to patients endorsing complex psychiatric issues [27]. Some argue, however, that distress or symptom exacerbation may be a key part of the therapeutic process and does not necessarily impact treatment effectiveness or drop-out rates [28,66].

In keeping with the general psychotherapy literature, indicators of therapeutic alliance and relational attunement were critical for engagement in EMDR and successful treatment outcomes [66]. Therapeutic attunement was reflected in the clinician’s empathetic awareness of, and responsiveness to, each participant’s unique emotional needs and moods, and provision of validation and acceptance of the participants’ states and experiences [67]. Clinician qualities enabling attunement include flexibility, intuition, empathy, ease in working with trauma, and attending to subtle gestures of care [68–70]. The clinician’s ability to attune and co-regulate allowed participants to feel safe, comfortable, and well-supported. This state of safety may set the conditions necessary for patients to have corrective emotional experiences and adaptive memory reconsolidation [71,72]. For complex psychiatric populations, relational attunement may be vital to trauma processing, particularly when patients are experiencing crises and are unable to effectively utilize their own coping skills [73]. The importance of attunement and secure therapeutic relationships have also been emphasized in EMDR and TFP research [26,39,70,74,75], best practice recommendations for treating individuals with dissociative identity disorder [69], and general discussions regarding the role of mental health therapists.

This study also adds to literature regarding potential for improving services for those with suicidal ideation. For example, participant suggestions mirror published literature suggesting benefits of individualized psychotherapeutic treatments for improving treatment response among individuals with PTSD, especially for more complex forms of trauma [65,76]. Study participants identified many unique needs and treatment preferences, such as readiness for treatment, the mode of EMDR delivery, and the frequency, intensity, and length of treatment. This variability may have, in part, stemmed from the unique combination of biological and psychological experiences that culminated in participants developing complex psychiatric issues, including SI. Understanding and incorporating these factors is an ongoing avenue of research [77].

The integration of qualitative research and patient feedback about lived experiences, as captured in this study, is crucial for the intentional co-production of healthcare services with the intent of improving treatment outcomes, particularly for complex or heterogenous clinical populations [44]. Co-production of healthcare services [78] involves viewing key stakeholders (e.g., patients) as empowered to take some control of the service agenda and assume some responsibility for improving care [79]. Such co-production of services may provide substantial benefits, including improvements in quality of service, effectiveness, and long-term cost effectiveness of treatments [80]. Further collaboration between patients, mental health clinicians, and healthcare policy makers may aid in reforming the current landscape of SI treatment.

### Strengths and limitations

The greatest strength of this study lies in addressing the notable gap in patient-focused research within the EMDR literature. This research is the first to capture the lived experiences of individuals with SI undergoing web-based EMDR. Qualitative findings may inform the future delivery of psychotherapeutic care for individuals experiencing SI. Finally, the inclusion of a diverse psychiatric population was another notable strength of this study.

Several important limitations must also be acknowledged. First, generalizability of results is limited by the study sample size and qualitative methodology. Treatment response reported in this study may have been impacted by several factors, including the unique experiences and history of individual participants, individualization of treatment, and specific qualities of study therapists. Further, individuals with severe dissociation and dissociative disorders were excluded from participation. We therefore caution against applying these findings to those with severe dissociation or dissociative disorders. Second, we were only able to interview 14 of the 20 eligible participants, and not all participants completed the 12-session EMDR treatment course, potentially introducing bias. Third, interviews were conducted at variable time intervals following completion of EMDR treatment. Standardizing the time between treatment completion and interview would have been preferable. However, this was not possible given that this qualitative study was created as an amendment of the parent clinical trial after data collection had begun. Fourth, we did not calculate the interrater reliability of the codes we extracted. Calculating interrater reliability, although not necessary for qualitative analysis, may aid in improving the transparency of the coding process, promote reflexivity and dialogue within research teams, and increase the trustworthiness of the analysis [81]. Finally, study population demographics were relatively uniform, with all participants being heterosexual individuals living in urban centers, limiting our ability to explore how other backgrounds and traits could impact their experiences with web-based EMDR. We also did not collect data regarding participant perceptions of suicide and SI [82] or help seeking for mental healthcare [83]. Future research into psychotherapy for SI ought to take these factors into consideration.

### Future directions

Many challenges remain unresolved, particularly around treatment individualization. Findings from this qualitative study highlight several factors that may influence treatment response, including individual readiness to engage in therapy, the mode of therapy delivery, and preferences regarding treatment duration, intensity, and frequency. To address these complexities, we recommend that future research focuses on how to address individual treatment readiness and preferences. Incorporating short interviews after EMDR sessions, for example, could help gather valuable insights into patient satisfaction and treatment experience. Research into ways in which life experiences may alter SI presentation and suicidal states may inform the development of more effective, targeted, and individualized care. Such studies are key to ensuring the long-term viability and sustainability of implementing EMDR as a therapeutic approach, particularly for addressing SI. By prioritizing patient-centered research, we can better align therapeutic approaches with individual preferences, ultimately improving outcomes and enhancing the accessibility and relevance of treatment.

## Conclusion

To our knowledge, this is the first study to explore patient experiences of receiving web-based, therapist-delivered EMDR focused on addressing SI rather than a specific psychiatric diagnosis. Participants reported notable reductions in SI and evidence of PTG following EMDR, and highlighted therapist attunement as a key for treatment success. This research also sheds light on the variety of participant preferences and needs, informing more personalized EMDR. Finally, this study provides encouraging preliminary evidence for the use of EMDR with patients experiencing SI and lays the foundation for future research.
